# Commentary: Developmental Constraints on Learning Artificial Grammars with Fixed, Flexible, and Free Word Order

**DOI:** 10.3389/fpsyg.2018.00276

**Published:** 2018-03-06

**Authors:** Aniello De Santo

**Affiliations:** Department of Linguistics, Stony Brook University, Stony Brook, NY, United States

**Keywords:** language learning, cognitive development, artificial grammars, formal language theory, complexity

A long standing hypothesis in linguistics is that typological generalizations can shed light on the nature of the cognitive constraints underlying language processing and acquisition. In this perspective, Nowak and Baggio (2017) address the question of whether human learning mechanisms are constrained in ways that reflect typologically attested (*possible*) or unattested (*impossible*) linguistic patterns (Moro et al., 2001; Moro, 2016).

Here, I show that the contrasts in Nowak and Baggio (2017) can be explained by language-theoretical characterizations of the stimuli, in line with a relatively recent research program focused on studying phonological generalizations from a mathematical perspective (Heinz, 2011a,b). The fundamental insight is that linguistic regularities that fall outside of certain complexity classes cannot be learned, due to computational properties reflecting implicit cognitive biases.

## Developmental constraints on learning

In order to test whether adults and children have different biases toward typologically plausible patterns, Nowak and Baggio (2017) construct 4 finite state grammars imposing varying constraints on word-order (fixed: FXO1 and FXO2; flexible: FLO; and free: FRO), instantiated over two word-classes: shorter, more frequent words (F-word) or longer, less frequent ones (C-words). Participants were asked to differentiate between strings produced by the grammar they had been trained on, and strings produced by a different grammar (e.g., FXO1 vs. FLO). Adults succeeded in recognizing fixed and flexible word-order strings (Experiment 1: FXO1 vs. FLO) and failed in recognizing free word-order strings (Experiment 2: FXO2 vs. FRO). In contrast, children could recognize flexible word-order and free word-order strings, but not fixed word-order strings (Experiment 3 and 4, replicating the contrasts of Experiment 1 and 2). The authors attribute these results to the inability of children to acquire *typologically implausible* grammars, suggesting that adults either have distinct constraints on language learning, or are able to employ more general learning strategies.

## Subregular complexity

Nowak and Baggio (2017) control for information-theoretical differences (e.g., Shannon entropy; Shannon, 1948) among strings to explicitly refute computational explanations of their results. Crucially, a different computational measure—based on language-theoretical characterizations sensitive to structural properties of the grammars—is dismissed by assuming that the finite-state grammars generating the stimuli lead to languages of equivalent complexity (i.e., regular languages).

This latter assumption is grounded in the Chomsky Hierarchy (Chomsky, 1956), which divides languages (*string-sets*) into nested regions of complexity (*classes*) based on the expressivity of the grammars generating them. However, while regular languages were originally treated as a monolithic unit, it has been shown that they can be decomposed into a finer-grained hierarchy of languages of decreasing complexity—the *Subregular Hierarchy* (McNaughton and Papert, 1971; Rogers et al., 2010). A case has been made for the relevance of this classification for cognition (Rogers and Pullum, 2011; Heinz and Idsardi, 2013; Rogers et al., 2013). Recently, it was posited that the complexity of human language patterns is bound by classes in this hierarchy (the *Subregular Hypothesis*; Heinz, 2010; McMullin, 2016; Graf, 2017), which have been shown to make valuable generalizations across different domains (Aksënova et al., 2016; Aksënova and De Santo, 2017). It also appears that the simpler classes in the hierarchy are more easily learnable by humans (Hwangbo, 2015; Lai, 2015; Avcu, 2017).

Here, my focus is on *Strictly k-Local* (SL_*k*_) languages, which define strings in terms of finite sets of allowed *k*-grams—contiguous sequences of symbols of length *k*. Consider *CFCFC* and *CFCFCC*, two well-formed strings for FLO. A strictly *k*-local grammar is constructed by listing the smallest set of *k*-grams needed to distinguish between well-formed and ill-formed strings (e.g., ^*^FCFCFC,^*^CFCFF):

FLO:={⋊C,CC,CF,FC,C⋉,F⋉}.

^1^

**Figure 1:**
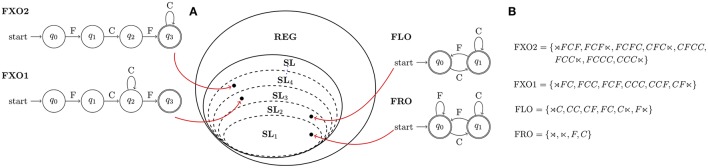
Nowak and Baggio (2017)'s artificial grammars **(A)** placed in the hierarchy of Strictly *k*-Local (SL_*k*_) Languages, and **(B)** their respective language-theoretical characterizations (⋊, ⋉ respectively mark left and right string-boundary); note that complexity decreases with subsumption, so *SL*_1_ ⊂ *SL*_2_ ⊂ *SL*_3_ ⊂ *SL*_4_ ⊂ … ⊂ *SL*_*k*_ implies FRO < FLO < FXO1 < FXO2.

Language complexity is measured not by the size of the grammar, but by the minimal length (*k*) of the substrings needed to generate *all (and only)* its well-formed strings. Thus, FLO is a Strictly 2-Local (SL_2_) language. Similarly, FRO is SL_1_, FXO1 is SL_3_, and FXO2 is SL_4_ (cf. Figure 1). Importantly, SL languages form a proper hierarchy in *k*: FRO is then the simplest language, while FXO2 is the most complex.

We can now interpret the learnability differences shown for adults vs. children, in light of the subregular complexity of the target string-sets. The contrast between FXO1 and FLO (Experiment 1 and 3) shows that SL grammars are equivalently easy for adults independently of the dimension of the *k*-grams; while children seem unable to correctly generalize over grammars with complexity greater than SL_2_. Language-theoretical considerations also allow for a deeper understanding of the contrast between FXO2 and FRO (Experiment 2 and 4). In Experiment 2, adults perform well when trained over FXO2: if adults can easily learn SL grammars of any size, this is not an unexpected result. What should come as a surprise is the low performance on FRO, the simplest SL_1_ grammar. However, consider that by construction FRO allows for any possible combination of symbols from the alphabet. Therefore, the set of strings generated by FXO2 is a proper subset of the set generated by FRO. Low performance of adults trained on FRO is then expected: since strings from FXO2 are also possible strings for FRO, participants will recognize every string as grammatical, and perform worse on the recognition task. Keeping in mind this possible confound, Experiment 4 (low accuracy when trained on FXO2 vs. FRO) suggests that children might be biased in favor of less restrictive and computationally simpler grammars.

## Concluding remarks

Nowak and Baggio (2017) present an interesting investigation of developmental biases in language learning mechanisms. I argue that a subregular characterization of their stimuli can help interpret learning differences between adults and children, thus suggesting that the *nature* of the observed biases is in fact intrinsically computational. From this perspective, *unlearnable* patterns would be those requiring computational resources that exceed what is allowed for a specific cognitive subdomain. What emerges is a strong parallel between language-theoretical approaches, and a research program focused on understanding *possible/impossible* patterns in human languages. Thus, as Jäger and Rogers (2012) suggest, closer collaborations between cognitive scientists and formal language theorists would improve the design and interpretation of artificial grammar experiments targeting human language biases.

## Author contributions

AD reviewed the literature, developed the theoretical stance, and wrote the manuscript.

### Conflict of interest statement

The author declares that the research was conducted in the absence of any commercial or financial relationships that could be construed as a potential conflict of interest. The reviewer CC and handling Editor declared their shared affiliation.

## Footnotes

^1^⋊, ⋉ mark left and right string-boundary.
